# Exploring the relationship between stigma and help‐seeking for mental illness in African‐descended faith communities in the UK

**DOI:** 10.1111/hex.12464

**Published:** 2016-04-28

**Authors:** Nadia Mantovani, Micol Pizzolati, Dawn Edge

**Affiliations:** ^1^ Population Health Research InstituteSt George's University of LondonLondonUK; ^2^ Department of EconomicsManagement, Society and InstitutionsUniversità del MoliseCampobassoItaly; ^3^ Centre for New Treatments & Understanding in Mental Health (CeNTrUM)Institute of Brain, Behaviour & Mental HealthThe University of ManchesterManchesterUK

**Keywords:** Black and minority ethnic groups, culture, faith based organisations, help‐seeking, mental illness, stigma, UK

## Abstract

**Background:**

Stigma related to mental illness affects all ethnic groups, contributing to the production and maintenance of mental illness and restricting access to care and support. However, stigma is especially prevalent in minority communities, thus potentially increasing ethnically based disparities. Little is known of the links between stigma and help‐seeking for mental illness in African‐descended populations in the UK.

**Objective and study design:**

Building on the evidence that faith‐based organizations (FBOs) can aid the development of effective public health strategies, this qualitative study used semi‐structured interviews with faith groups to explore the complex ways in which stigma influences help‐seeking for mental illness in African‐descended communities. A thematic approach to data analysis was applied to the entire data set.

**Setting and participants:**

Twenty‐six men and women who had varying levels of involvement with Christian FBOs in south London were interviewed (e.g. six faith leaders, thirteen ‘active members’ and seven ‘regular attendees’).

**Results:**

Key factors influencing help‐seeking behaviour were as follows: beliefs about the causes of mental illness; ‘silencing’ of mental illness resulting from heightened levels of ideological stigma; and stigma (re)production and maintenance at community level. Individuals with a diagnosis of mental illness were likely to experience a triple jeopardy in terms of stigma.

**Discussion and conclusion:**

‘One‐size‐fits‐all’ approaches cannot effectively meet the needs of diverse populations. To ensure that services are more congruent with their needs, health and care organizations should enable service users, families and community members to become active creators of interventions to remove barriers to help‐seeking for mental illness.

## Introduction

People with mental illness experience more stigma than those with other health problems.^1^ In part, this is due to the fact that people's general knowledge of mental illness is very poor despite the increasing availability of information.^2^ Reducing the stigma associated with mental illness has become an area of increased effort and attention worldwide.^3^, ^4^, ^5^ In the UK, stigma reduction has become a public health priority following the publication of a number of policy documents,^6^, ^7^ where challenging stigma is cited as a fundamental strategy to achieve social justice and equity.^7^

### Mental illness in minority groups

African‐descended groups in the UK (primarily people with origins in Africa and the Caribbean) experience poorer outcomes against a number of key social and health indicators.^8^, ^9^, ^10^, ^11^, ^12^ They are more likely to be diagnosed with psychotic illnesses and are over‐represented in inpatient psychiatric services.^13^ There is also evidence that they are confronted with ethnically based prejudice and discrimination by health professionals.^14^ These factors partly explain low levels of engagement between members of these communities and mainstream mental health services, access through adverse pathways, poorer experiences and outcomes.^15^ Delayed and non‐engagement have been consistently reported in the UK in relation to inferior access to mental health services by African‐descended people, resulting in longer duration of untreated illness, which is associated with more severe and chronic presentation at contact with services.^15^ Alongside mistrust of services, stigma is implicated in delayed help‐seeking.^16^ Stigma emanating from and reinforced within ethnic communities and families was reported by Anglin *et al*.^17^ whose study identified and linked stigmatizing attitudes to messages about mental illness communicated by family.

### Stigma

Stigma is principally a psychological and social phenomenon. Link & Phelan^18^ defined stigma as the process whereby labelling, stereotyping, separation, status loss and discrimination co‐occur in the context of power. These components can then lead to devaluation, rejection and exclusion of certain groups, which contributes to social disadvantage and loss of social status.^19^

Stigma occurs at three interacting and mutually reinforcing levels: self, social and structural.^20^, ^21^, ^22^ Self‐stigma involves the perceptions and experiences of those who possess stigmatized attributes. Individuals with stereotyped characteristics, such as mental illness, are frequently socialized into believing that they are devalued members of society, which leads to adopting negative feelings about self, engaging in maladaptive behaviour, and identity transformation^23^ such as feeling of shame and reduced self‐efficacy.^24^ Felt and perceived stigma, Scambler^25^ argues, is the internalized sense of shame and immobilizing anticipation of enacted stigma – the discrimination by others on grounds of being imperfect.

Social stigma refers to community members adjudicating particular traits to be contrary to community norms and behaving in a harmful way towards individuals who possess the devalued attribute.^20^, ^26^ Social stigma is produced by the individual and collective beliefs of dominant members of society, from which acceptable ways of behaving towards oppressed groups are defined,^27^ and is expressed behaviourally during interpersonal interactions.^18^ It provides fertile ground for the production of self‐ and structural stigma.^19^ For instance, the belief that all people with mental illness are dangerous may disincline some people to befriend anyone with a mental illness (contributing to self‐stigma) and may be supportive of coercive mental health interventions (contributing to structural stigma). Although the three levels of stigma are inextricably linked, this study principally focuses on personal and social stigma.

The profound impact of stigma on mental illness is well researched.^5^, ^28^ However, little is known of the relationship between stigma and help‐seeking among Black and ethnic minority (BAME) groups in the UK. This evidence gap requires urgent attention especially given the reported high prevalence of serious mental illness such as psychoses and schizophrenia among African‐descended groups^15^ coupled with markedly high levels of stigma against mental illness in these communities.^29^ Research conducted with African Caribbean groups suggests that religious beliefs and practices may amplify the processes of construction and reproduction of stigma.^30^

The overall aim of this study was to explore the factors involved in the social production and practice of stigma among African‐descended communities with a specific focus on elucidating the ideas, values and understandings that people deploy within specific social networks and how these impact help‐seeking for mental illness from/and engagement with formal mental health services.

## Methods

This study explores stigma associated with mental illness among faith‐based African‐descended communities in south London, locating the narratives of 26 interviewees within an interpretative framework constructed on the basis of the reading of interdisciplinary literature on stigma. The study employed an interpretative design, reflecting our interest in sense‐making and on gaining insight into the complex relationship between mental illness, stigma and help‐seeking. Of particular interest was understanding the social production and practice of stigma in African‐descended communities and its effect on help‐seeking.

### Sampling and recruitment

We applied a purposive convenience sampling strategy to recruitment. Specifically, we drew participants from an existing sampling frame of African‐descended individuals taking part in an outreach intervention programme focused on promoting mental health in minority communities.^31^, ^32^ The larger evaluation study directly involved community members in the delivery of the pilot project, which espoused an empowerment model whereby the local groups identified mental health needs, and mobilized themselves into action to address the recognized inequalities through employing and training Community Well Being Champions.^31^, ^32^ To obtain maximum variation within the sample, participants were selected to reflect key demographic characteristics such as gender, age range, ethnicity, literacy, understanding of English and role within the FBOs (faith leaders = FL; active members engaged in outreach work = AM; and regular attendees = RA). Of the 35 people we approached, 26 agreed to participate (8FL; 14AM; 13RA).

We chose to recruit our sample from FBOs taking part in the intervention because of the focus of our study and previously reported difficulties in recruiting from ‘hard‐to‐reach’ communities.^33^ Participants were recruited via eight geographically adjacent FBOs of differing Christian denominations. The study was conducted in London in one south Local Authority identified for its geographical diversity along with high levels of unmet mental health needs in its African‐descended communities.^34^ The programme's organizers from African Caribbean communities facilitated the recruitment process; they codesigned the information material explaining the purpose of the study and the ethics of participation, and devised and distributed the invitation letter to identified participants.

### Data collection and analysis

Data were collected between August 2012 and July 2013 via semi‐structured interviews lasting between 45 and 60 min and recorded with the consent of the respondents. The topic guide was embedded in the literature on mental health in BAME groups. Although alternatives were offered, all participants chose to be interviewed in the FBOs to which they were linked.

All interviews were transcribed verbatim and transcripts were uploaded into NVivo qualitative data analysis software version 10.^35^ Thematic analysis was adopted to identify, analyse and report patterns (themes) within data.^36^ The analysis consisted of four iterative phases where NM and MP (i) read the entire data set searching for meanings and patterns; (ii) generated initial codes through labelling features of the data; (iii) inspected the data for themes which involved reviewing the coded data to identify areas of similarity and overlap between codes; and (iv) produced a thematic map applied to the entire data set and the themes coded in earlier phase (quality checking). We applied Lincoln and Guba's^37^ idea of credibility to increase internal validity through developing early familiarity with the culture of the participating FBOs before data collection which entailed a ‘prolonged engagement’ with community members engaged in the outreach intervention.

### Ethics

The data collection for this aspect of the study fell under the remit of the aforementioned study.^31^ The pilot service, which was coproduced in partnership with community organizations and South West London Mental Health Trust, did not entail any change to the standard service being delivered (e.g. no randomization of service users into different groups); hence, ethical approval was not required. Nevertheless, all procedures contributing to this work complied with the ethical standards of National and Institutional Committees on human experimentation and with the Helsinki Declaration of 1975, as revised in 2013,^38^ and the Data Protection Act (1998).^39^

## Results

A total of 26 interviews were completed with 14 African Caribbean and 12 African participants. Five were British born, eight were born in the African continent, and 13 were born in the Caribbean Islands. Six were faith leaders (five males and one female), 13 congregants engaged in outreach work (seven males and six females) and seven congregants attending mental health awareness events (two males and five females). Participants were aged between 24 and 75 years.

Three key themes emerged from the data which are illustrated by direct quotes related to the following: the sociocultural beliefs about mental illness and the production of stigma, the social consequences of stigma mental illness, the impact of avoidance behaviour on help‐seeking and the reproduction of stigma in faith communities.

### Sociocultural beliefs about mental illness and the production of stigma

Participants’ accounts of mental illness neither conformed to a medical model of seeing mental health problems as forms of illness nor a continuum model with different degrees of mental health and illness. Instead, some spoke of ‘insanity’ or ‘madness’ which they associated with violence and danger. We extracted two broad views of mental illness from the data; one embedded in socio‐Christian beliefs about demon possession, devil and evil spirits, and the other rooted in non‐normative behaviour as illustrated by these quotes.

Those of us from Black background and some of us who were brought up outside of this country, our perception of mental illness is somebody's totally derailed and is walking in the street probably naked. It's somebody who is just a psychiatric inefficient. That's our understanding of mental illness, and so if anybody tells you that you have a mental health issue you are ready to fight them for saying that. I mean… because we relate mental health to insanity, a total level of insanity (FL05, African).

Mental illness, as somebody who comes from Africa, we think it's a curse. We think you're possessed by the devil, but it's a mental health problem. We don't know that. So it's a kind of a stigma that we Black people are taught; people with mental illness who are not the same [as us], who are not completely normal as we are (AM03, African).

A view expressed particularly among faith leaders was that mental illness was indicative of a moral failing on the part of the individual. For instance, FL02 (African Caribbean) said that African‐descended people ‘are supposed to be spiritually strong’. Profound belief in moral fortitude in the face of adversity meant that mental illness was regarded as a phenomenon experienced by those who failed to conform to societal norms. According to participants, such beliefs were likely to generate stigma towards individuals with mental illness.

Mental illness [in our communities] is seen as weakness and we don't handle weakness very well. Particularly in church, we don't want to be associated with weakness…so, if one is ‘weak’ then there's something wrong with you, and if there's something wrong with you then somehow you are less than [deficient]. You don't have enough faith. You don't have enough belief in God. There's not enough of God in you because if there was enough of God in you, you wouldn't be here at this particular spot now (FL06, African Caribbean).

The conceptualization of mental illness as a moral failing or weakness was considered problematic in terms of help‐seeking because it did not command the same attention or level of empathic understanding as physical illnesses.

Mental illness is not given the appropriate attention as, say, maybe a broken arm or high blood pressure. It's not given that same level of attention because it's seen as weakness and we don't handle weakness very well (FL06, African Caribbean).

There was some disagreement among participants as to whether attitudes towards mental illness differed among people born in the UK vs. Africa or the Caribbean. For some respondents, acculturation seemed to change the ways in which people perceived mental illness, whereas others were puzzled at the lack of change over time.

The stigma [attached to mental illness] comes in two perspectives….for those of us who are brought up in Africa, it's different from those who are born here and born into a family where everybody in their family probably was also born here. It's different. They are also in denial of accepting that ‘my child is having mental health issues’. Instead of them addressing it, they would rather look at it from a different point of view (FL05, African).

…what I still can't understand is how is it that people, youngsters who were born here, are still affected by it. They still cannot accept that, you know, ‘Oh, I've got an uncle who has got a problem.’ It's fear and stigma and a kind of self‐loathing. I think these three things have bedeviled the Black community. When I speak to people, I'm amazed how it comes across (AM01, African Caribbean).

### Social consequences of stigma mental illness

The perceived social consequence of stigma associated with mental illness and the interrelationship between social and individuals’ personal (internalized) stigma is described. This extract illustrates respondents’ perceptions of the complex ways in which social stigma towards mental illness affects the individual both in terms of curtailed opportunities and disengagement from mental health services and their communities.

You are rejected by your own community, by your own environment. They will say that you're not useful any more. Stigma affects [people] individually in terms of denying things. I mean as a victim, there's feelings of being kind of being hurt, being ostracised, being isolated and there's also the case of not seeking appropriate help, not engaging with the services available to actually deal with something properly. The biggest damage is the person's attitude to themselves, in terms of what they're going through and also how that influences what they do to try and deal with their situation (AM04, African).

Loss of aspirations was regarded as being the direct result of the negative effects of internalized stigma related to mental illness. Moreover, respondents believed that loss of aspirations was often reinforced by employers’ discriminatory practices towards people with mental illness, especially African‐descended people. Respondents pointed out that marks of difference (psychiatric labelling, hospitalization, overt symptoms such as disordered thinking and visible side‐effects of medication) stained the person indefinitely irrespective of the degree of recovery.

For some of the people I have spoken to, it [mental illness] sort of cuts short any types of aspirations and a hope that they may have had …it cuts short any type of opportunities, any type of beliefs anybody would have in them. You receive a label of mental ill health, anything that you do, whether you are feeling well or unwell, you will be pathologised (RA04, African).

Participants described the response and the sense of shame (internalized stigma) experienced as a result of having someone in the family diagnosed with a mental illness. The extracts below highlight stigmatizing attitudes that exist in African‐descended families, underscored by the fear of becoming ‘contaminated’ as a result of association with someone with mental illness:

If somebody within their family has gone mentally ill, it's a shame, and they rather push that person out of the way and don't talk. So, you have that thing that you bring that kind of stigma with you. If somebody goes mentally ill in your family you don't talk about it really. If it goes wrong in a family, something is wrong with that [whole] family, so, you shut that person away. It's this big, denial, and it has been from the whole cultural thing (AM06, African Caribbean).

Denial and refusal to accept someone in the family with mental illness was thought to be underpinned by a lack of understanding about mental illness. In their accounts, participants indicated that families they knew would go to the extent of avoiding contact with someone in the family with a mental illness, resulting in family withdrawal and increased social isolation especially when persons were hospitalized.

In some ways, [if you are] mentally ill you are isolated in the hospital and that's it. Even sometimes families wouldn't go and visit because they don't want to know. In a way, we kind of try and bury it, it's not happened to us. It's a sense of denial that this has not happened, so, we deny it. We're not open with it and we don't want to talk about it. It's the shame that somebody within their family has gone mentally ill (RA07, African).

In the eyes of participants, mental illness reflects badly on the whole family; thus, strenuous efforts are taken to avoid disclosing family members’ mental illness to maintain an ‘idealized social identity’. This was especially so, if ‘you're somebody who has influence or weight in the community, as you would not like it to be known that there's something wrong with you, and be associated with weakness’ (FL06, African Caribbean). In this respect, a participant commented:

There's a lot of ideological stigma in mental illness as well as a lot of shame in terms of how this makes you look in front of other people. It is shame because of what it means in the eyes of the family, in the eyes of the community, because as a people, our family and our community kind of mean a lot to us (AM04, African).

### ‘Silencing’ mental illness and avoidance behaviour impacting on help‐seeking

‘Silencing’ mental illness through denying affiliation with a ‘spoiled identity’^40^ and avoiding talking about it for fear of potential repercussions to the family's reputation was imputed as a barrier to seeking help from mental health services. The fact that mental illness in these communities is kept ‘as a secret and people will not even go to the doctor’ (FL03, African Caribbean), was implicated in deteriorating mental health and more severe symptoms by the time African‐descended individuals come into contact with mainstream mental health services.

Even within the family, when they have it [mental illness], they won't let people know that a member of their family is having that, and as a result it escalates and results in them rather than being treated, it wasn't a situation they want to face [they deny it]. That's the key thing, if they don't come out. People keep it to themselves and the way they see mental illness it's something very, very terrible and it's: ‘God forbid it's not my child’ (AM12, African).

In this sample, an important by‐product of family's behavioural response to feelings of shame was lack of parental support. According to respondents, this impacted on the person diagnosed with mental illness in terms of self‐efficacy; ‘not seeking help as early as it should and wait for the situation to become worse’ (AM13, African Caribbean). This is illustrated in this account of how a family's distancing from and denial of their son's mental illness enacted stigma, provoking a feeling of shame and a sense of emotional separation and isolation from his own family:

I remember when I went to see someone and his family was around him and I was speaking with him and the family was saying: ‘Could you please help him because we don't have this in our family? He needs to pull himself together. He needs to sort himself out.’ And you could see how this person felt ashamed and was becoming more and more isolated within himself [….] They're not surrounded by family or friends anymore because they don't want people to know that they've got this illness, so they become quite lonely (FL04, African Caribbean).

Respondents suggested the interrelationship between personal (internalized) and community‐level stigma was an important factor in delayed or non‐engagement with mental health services. According to some participants, ‘African and African‐Caribbean communities were not as engaged as we could be’, thus making people with mental illness more vulnerable to ‘some of the things they experienced around stigma’ (AM02, African). Indeed, several participants stated that the stigma attached to mental illness was more important than the illness itself, which represents a significant barrier to disclosure and help‐seeking:

So, I think what that causes … it stops the community from talking about these issues because of the stigma and they don't want to be associated or they don't want no‐one to look at them and say: ‘Oh, this person is crazy,’ because they mentioned the word mental illness and what not. So, I think we protect ourselves by avoiding having to talk about or say any of those things (AM02, African).

So powerful is the stigma of mental illness in these communities that it was regarded by some respondents as a form of demonization, which was perceived as detrimental both to the individual experiencing mental illness and the communities as a whole. Such demonization led to lack of discourse – a ‘silencing’ about mental illness, which negatively impacts on communication between families, and most importantly, between community structures and services designed to provide care and support.

I think with mental health issues there has been a longstanding demonisation of anything of people that don't fit. There hasn't been an extended hand to help them. And people might withdraw from that individual instead of seeking to help them. It's divisive, it divides the community. It leaves people who are suffering from mental health issues isolated, being treated by… unfamiliar group of people [mental health professionals] that don't understand their culture, their values or their norms (RA03, African).

It breaks down communities in terms of communication. It develops – people are isolated. It's almost self‐perpetuating; a self‐perpetuating snowball. The more it goes on the less we talk, the less we talk the more it goes on (FL06, African Caribbean).

### The reproduction of stigma in faith communities impeding/delaying help‐seeking

Participants revealed that the church was often the first port of call when someone experienced psychological distress in their communities and that church leaders often adopted primarily spiritual approaches to providing help and support rather than advising them to seek help from mental health professionals. In this context, some respondents were critical of practices such as praying for those who had mental health concerns, or asking them to fast, or performing exorcism as they believed these practices were likely to impede help‐seeking from mental health services with potentially deleterious consequences for the mental health of those individuals seeking help solely from FBOs.

If somebody comes into the church with a mental health issue, they [pastors] are most likely to pray for this person and annoy the person with asking them to try things, rather than asking the person to seek for professional help […] Pastors think a person taken over by an evil spirit has mental illness, so, the evil spirit must be exorcised out of them. And once you exorcise people they do not progress to become better, they progressively become worse (AM03, African).

Respondents reported that purely spiritually based approaches to dealing with mental health needs denoted pastors’ lack of understanding of mental illness. One faith leader observed that ‘[M]any of us are praying from the level of ignorance’ (FL05, African) and that pastors were often unaware of their lack of knowledge around mental illness. However, this was not reflected in all faith leaders’ accounts among whom there was a recurring theme of recognizing that they were not equipped to assess the presenting symptoms of those seeking help from them, and therefore unable to respond in ways that would facilitate help‐seeking and engagement with mental health services and/or reduce stigma.

We do believe in spirits’ influence on people's minds and behaviour but not in everything. Not all behaviour is necessarily a spiritual manifestation, and I don't think we always make that distinction. And in many cases we're not equipped to distinguish what is spiritual need and what is mental health need. I think we mix them up and so everything becomes demonised and it reinforces the stigma, ‘the person with the thing’, you know (FL02, African Caribbean).

## Discussion

African‐descended communities in the UK have reportedly high rates of diagnosed serious mental illness such as psychosis.^41^ Nevertheless, their engagement with mental health services and access to care and treatment is consistently reported as suboptimal and members of this ethnic group are often labelled ‘hard‐to‐reach’.^42^ Research also indicates high levels of stigma in this and other BAME communities.^16^, ^17^ In this study, we sought to understand, from community members’ perspectives, how and to what extent personal and social stigma influence help‐seeking from mainstream provision [National Health Service (NHS)] in particular. This study utilized qualitative interviews with African‐descended individuals living in south London who were faith leaders, active members engaged in outreach work and regular attendees of Christian churches. It was located within FBOs because previous UK research has suggested that Black‐majority churches (BMCs) are important sources of help and support from which community members (irrespective of religious affiliation) often seek help for mental health problems, in preference to more formal structures such as the NHS.^43^, ^44^

Our findings illustrate the cultural context and beliefs about mental illness underscoring its close connection to help‐seeking, which is the by‐product of complex interplay of social, cultural and personal factors overlaid by high levels of stigma associated with mental illness. Factors contributing to stigma that were obstacles to help‐seeking from mental health services were as follows: cultural beliefs about mental illness; practices in faith communities; anticipation/experience of negative consequences; family kinship/relational structure; and preference for non‐disclosure. Public attitudes about mental illness in the English‐speaking Caribbean is characterized by high levels of stigma^45^ which lead individuals to turn to religious leaders or to engage in religious coping rather than seeking psychiatric/psychological help for mental health problems.^46^, ^47^ Similarly, in East and West Africa, traditional healers are usually the primary source from which people seek help, advice and care when faced with mental health problems. Frequently, this is the only source of care sought.^48^, ^49^, ^50^ In line with our findings, Nsereko *et al*. reported that cultural perceptions of mental disorders as ‘spiritual’ illnesses^48^ can profoundly influence where and whether help is sought. These barriers to help‐seeking are further compounded by the orientation of orthodox psychiatric services, which may be alienating and foreign for people with alternative beliefs and worldviews.^51^

In our study, beliefs about the relationship between evil spirits, devil possession and mental illness carried with them implicit assumptions about moral failings on the part of individuals experiencing psychological distress. It is likely that, within African‐descended communities, this finding relates not only to serious mental illnesses which are sometimes linked with violence in the minds of the general public but extends to more common mental disorders.^43^, ^44^ Social psychiatry suggests that cultural beliefs play an important role in shaping societal responses to people with mental illnesses, influencing stereotyping, service provision and help‐seeking.^52^

The interaction between faith and kinship/relational structures was a factor in creating and perpetuating interpersonal stigma. The effect might be described in relation to Serrant‐Green's^53^ ‘screaming silences’ framework. Although originally developed in relation to physical illnesses, evidence from this study and elsewhere suggests the framework (which describes how individuals suffer in silence because of shame, stigma and related absence of discourse within these communities) is applicable to mental illness.

Given community members’ tendency to turn to the church for help in times of trouble, faith leaders’ reports of their lack of expertise in relation to mental illness is an important finding. As reported by respondents, normative cultural beliefs in the existence of evil spirits and demonic possession might influence perceptions of what may (or may not) be evidence of psychiatric illness. Moreover, the Black‐majority church's ‘symbolic centrality and historic multifunctionality’^54^ ^: 177^ might explain the preference for community‐level alternatives (such as local pastors or folk practitioners) vs. mainstream sources of help in African‐descended communities,^55^, ^56^ which could impede affected individuals’ engagement with formal mental health services. The significance of our study is that, as with findings from a north of England sample of Caribbean‐origin women,^43^ these attitudes and help‐seeking preferences have persisted among subsequent generations of British‐born people of African descent.

Heightened anticipation/experience of negative consequences resulting from a diagnosis of mental illness in the family, which is to be kept behind closed doors,^57^ may be partly explained by the collectivist nature of African‐descended cultures,^58^, ^59^ and by the ideological stigma attached to family honour or ‘good name’. In this context, mental illness can be seen not solely as negatively impacting the individual but also as ‘contaminating’ the extended family and potentially the entire community. Under these circumstances, our findings indicate that individuals diagnosed with mental illness are likely to experience a triple jeopardy in terms of stigma – rejection by their families, stigma and alienation from their communities and internalized ‘self‐stigma’. In consequence, they are at risk of increased social isolation which is both antithetical to recovery from mental illness and increases the likelihood of relapse and hospitalization,^60^ which further reinforces stigma.

Our findings suggest that to tackle ethnically based disparities in mental health and to provide appropriate and responsive mental health services that meet the needs of a diverse population, key factors such as race/ethnicity, faith and culture need to be taken into consideration as they all affect how mental illness is perceived, experienced and managed. The analysis of the aforementioned axes of diversity offered a nuanced understanding of stigma associated with mental illness and its effect on help‐seeking.

Public sector resources have often failed to meet the needs of persons with mental illness, especially BAME groups,^61^ who are less likely than White people to seek treatment from mainstream sources.^62^ It is apparent that ‘one‐size‐fits‐all’ approaches cannot effectively meet the needs of diverse populations. The provision of appropriate and responsive services that meet the needs of people experiencing mental illness should take account of intersectional complexity and attempt to develop culturally sensitive services and therapies in partnership with those who will become the recipients. Commissioners and providers of mental health services need to work with service users, families and community members to become cocreators of interventions and providers of solutions to remove barriers to help‐seeking for mental illness. FBOs are trusted entities within many communities with histories of providing spiritual refuge and renewal. They have the potential to become bridges between the cultures of health care and different minority communities and thus bring about change by improving communication and information sharing.^31^, ^32^ However, the failure of commissioners and service providers to establish effective partnerships with community groups means that these organizations remain an underutilized resource for tackling ethnically based mental health disparities.

### Strengths and limitations

Adopting a qualitative approach enabled us to construct a nuanced, detailed picture of the relationship between stigma, help‐seeking and axes of diversity. However, this methodology has limitations – the sample size is modest and cannot be assumed to be representative of African‐descended communities. Due to time and financial limitations, it was not possible to interview the stigmatized persons or non‐FBOs individuals. We acknowledge that these are important additional perspectives if we are fully to understand the impact of stigma on individuals experiencing mental illness. Despite these limitations, this study yields important insights into the intersections between stigma, faith and mental illness within African‐descended communities in the UK, highlighting implications for help‐seeking.

The study provides corroboration and further understanding of the role of stigma as a barrier to accessing care for mental illness, which disproportionally impacts help‐seeking among people of BAME backgrounds.^63^ The aforementioned themes extend existing models about how stigma reduces help‐seeking^20^, ^64^ and how the deterrent effect of stigma on help‐seeking can be counteracted.^63^ Nevertheless, diaspora approach to further research (exploring similarities and differences between migrant African‐descended people and those in their home countries) ought to be undertaken to test the empirical evidence reported here on the processes of stigma and its effect on help‐seeking. A further line of enquiry could be assessing the extent to which Islamic or other FBOs perspectives concur with those from Christian FBOs. This could have important implications for commissioning and delivery of culturally sensitive mental health care – an approach analogous to gender‐sensitive care, which makes conscious attempt to acknowledge and take into consideration people's cultural backgrounds, beliefs and attitudes in the delivery of mental health care.

## Supporting information


**Appendix S1.** Project title: Stigma associated with mental illness in African and African‐Caribbean groups linked to FBOs.
