# Supplementary material for: Exploring the relationship between stigma and help‐seeking for mental illness in African‐descended faith communities in the UK
Source: Health Expect. 2016 Apr 28;20(3):373–84. doi: 10.1111/hex.12464 (PMC5433535; doi:10.1111/hex.12464)
Supplement: Supplementary file 1 — Appendix S1. Project title: Stigma associated with mental illness in African and African‐Caribbean groups linked to FBOs. [file HEX-20-373-s001.docx]

**Appendix:**

**Project title: Stigma associated with mental illness in African and African-Caribbean groups linked to FBOs.**

I am interested in find out about the perceptions of mental illness among African and African-Caribbean groups and how stigma associated with mental illness may affect their ability to seek help from mental health services.

I am going to ask you a number of questions. There are no right or wrong answers. I am interested in what you think. Please tell me as little or as much as you feel comfortable doing.

To help me remember what we discussed, I’d like to record our conversation with your permission. As I mentioned earlier [consent process], only members of the research team will hear the interview. Once I have typed up the interview, I will delete it. I’ll also take some notes. These are to help jog my memory. Is that ok?

1. What in your view people in your community commonly think when they hear the word ‘mental illness’ [probe whether this is a share belief among African and African-Caribbean groups]
2. There is a lot of talk about how mental illness can be stigmatising to people who are suffering from mental illness – can you tell me what is stigma for you? [probe how stigma related to mental illness can affect African and African-Caribbean groups]
3. I am interested to find out from you is: what can be done to ensure that people with mental illness are not stigmatized [probe how can stigma associated to mental illness be reduced in their communities]
4. It is known that people of African and African-Caribbean background do not go to their doctors or the local mental health services when they have a mental health problem – what is your view on this? [probe about why this is happening; and how can this be overcome]
5. Do you think the Black church could have a role in helping African and African-Caribbean communities in accessing mental health services? [probe how the church could help in improving these communities access to services -]

Is there anything else you would like to add about stigma and/or mental illness in African and African Caribbean-communities that we haven’t covered you think is important?
